# Phenotypically plastic drug‐resistant chronic myeloid leukaemia cell line displays enhanced cellular dynamics in a zebrafish xenograft model

**DOI:** 10.1111/jcmm.70105

**Published:** 2024-10-11

**Authors:** Seda Baykal, Zeynep Yuce, Gunes Ozhan

**Affiliations:** ^1^ Department of Molecular Biology and Genetics Izmir Institute of Technology Gulbahce, Urla Izmir Turkey; ^2^ Izmir Biomedicine and Genome Center (IBG) Dokuz Eylul University Health Campus Izmir İnciralti Turkey; ^3^ Department of Medical Biology Dokuz Eylul University Medical School Izmir İnciralti Turkey

**Keywords:** cell motility, cell plasticity, CML, invasion, K562, resistance

## Abstract

Understanding the mechanisms by which cancer cells switch between different adaptive states and evade therapeutic interventions is essential for clinical management. In this study, the in vivo cellular dynamics of a new chronic myeloid leukaemia cell line displaying altered phenotype and resistance to tyrosine kinase inhibitors were investigated in correlation with their parental cells for invasiveness/metastasis, angiogenic potential and population kinetics. We showed that the cells exhibiting drug resistance and plastic phenotype possess an increased capacity for invasion compared to their parental cells, that exposure to imatinib mesylate has the potential to enhance cellular motility and that in a leukaemic cell population, even a minority of plastic cells exhibit improved migratory ability. Furthermore, we show that these plastic cells have angiogenic and extravasation potential. The present study provides significant insights into the cellular dynamics displayed by a TKI‐resistant, phenotypically plastic CML cell line, using a zebrafish (*Danio rerio*) xenograft model.

## 1 INTRODUCTION

In cancer cell biology, phenotypic plasticity refers to the ability of cancer cells to change their characteristics and behaviour in response to different environmental cues. This plasticity is a major contributing factor to the heterogeneity and complexity of tumours and a predominant prerequisites of metastasis.^1^ Phenotypic plasticity in cancer cells also contributes to therapeutic resistance. Cancer cells that undergo phenotypic changes often exhibit resistance to chemotherapy and targeted therapy.^2^ This resistance arises from the altered signalling pathways and adaptation to changing selection pressures and competitive interactions in the microenvironment. Adaptation by phenotypic plasticity or an adaptive phenotypic shift (APS) is achieved by changing the transcriptional landscape. When challenged by an environmental insult the cancer cell is in a search of alterations in the expression of genes required to change its epigenetic landscape.^3^, ^4^ This landscape is a dynamic structure and offers acquired transcriptional flexibility as a stress response, allowing for a unique resistance model. Understanding the mechanisms by which cancer cells switch between different adaptive states and evade therapeutic interventions is essential for clinical management and prognostic assessments. Unfortunately, the biological processes involved are still poorly understood. Although there are many reports on the molecular mechanisms involved in cancer cell plasticity—especially under the context of epithelial‐mesenchymal transition (EMT), studies that report intercellular dynamics are relatively less predominant. In this study we aimed to examine the dynamics of phenotypically plastic and non‐plastic cancer cells both separately and in a mixed population.

Previously, we reported an APS as a mechanism for tyrosine kinase inhibitor (TKI) resistance in the chronic myeloid leukaemia (CML) cell line K562.^4^ CML is a haematopoietic stem cell disorder caused by the breakpoint cluster region protein (BCR)/Abelson murine leukaemia viral oncogene homologue 1 (ABL) chimeric oncogene. The BCR/Abl oncoprotein has aberrant tyrosine kinase activity and provides survival signals to malignant cells that drive the disease in terms of cell proliferation and resistance to programmed cell death.^5^, ^6^ Despite being highly resistant to conventional therapies, CML cells are sensitive to blocking the survival signal provided by BCR/Abl. The introduction of the TKI imatinib mesylate (IM) has redefined the treatment of CML. Nevertheless, some patients experience relapse and are resistant to imatinib.^7^, ^8^, ^9^ Defined TKI resistance mechanisms are far from covering all cases of TKI‐unresponsive CML patients and in many cases the cause of resistance remains unknown.^10^ In our previous study, we generated an IM‐resistant subpopulation of K562 cells with a new semi‐adherent phenotype called K562‐IR.^4^ We found that K562‐IR cells were resistant to not only imatinib but also several other TKIs including nilotinib, dasatinib, bosutinib and ponatinib, proliferated more slowly and had a semi‐adherent phenotype. Moreover K562‐IR cells displayed downregulated cell death signals, upregulated embryonic surface markers, re‐regulate genes involved in cell‐tissue‐organ differentiation and could form tumour spheroids. Thus, the resistant cells appeared to develop transcriptional instability, allowing them to adapt to environmental stress and resulting in APS. Although K562‐IR cells exhibit some leukaemic stem cell‐like properties, they, like their parental K562 cells, are CD34 negative. The TKI resistance observed in K562‐IR cells is not due to any known mechanisms.^9^, ^10^ These cells are Bcr‐Abl‐independent, no longer relying on it for survival. Phenotypically, K562‐IR cells are highly adherent and exhibit characteristics of partially reprogrammed cells. Cell surface marker and protein analyses indicate an adaptive phenotypic shift that confers drug resistance.^4^ This demonstrates that targeting driver mutations alone may not be sufficient to eliminate cancer cells, as their inherent plasticity can lead to phenotypic adaptations resulting in acquired resistance, independent of secondary genetic events. K562‐IR cells serve as a model for therapy resistance resulting from phenotypic shifting and potentially for dormant cancer cells that may cause relapse years after therapy. To study the cellular dynamics of the phenotypically plastic K562‐IR cells and the non‐plastic K562 cells, we employed in vivo techniques.

Zebrafish have become a widely used model organism due to their ability to be easily and inexpensively maintained, their high reproductive output, their rapid and transparent embryonic development and their suitability for various techniques such as transgenesis, live cell imaging, cell lineage tracing and micromanipulation.^11^, ^12^, ^13^ Zebrafish are particularly useful for cancer research, as the xenotransplantation of human cancer cells into zebrafish allows for the in vivo evaluation of cancer progression and drug discovery at a single‐cell resolution in real‐time. A variety of cancer models have been established using zebrafish larvae and adults in the literature.^14^, ^15^, ^16^, ^17^, ^18^ The zebrafish larval model is used in this study to investigate the behaviour of phenotypically adherent, TKI‐resistant K562‐IR cells in terms of invasiveness/metastasis, angiogenic potential and population kinetics.

## 2 MATERIALS AND METHODS

### 2.1 Cell culture

Human CML blastic phase cell line K562 and its IM‐resistant subpopulation K562‐IR were obtained as described in our previous studies.^4^ Cells were cultured in RPMI 1640 supplemented with 10% fetal bovine serum (FBS), 1 unit/mL penicillin G and 1 mg/mL streptomycin at 37°C and 5% CO_2_. K562‐IR cells were continuously cultured with 10 μM imatinib mesylate.

### 2.2 Zebrafish husbandry

Casper mutant (roy−/−, nacre−/−) and Tg(fli1:EGFP) transgenic zebrafish lines were supplied by Izmir Biomedicine and Genome Center (IBG) Zebrafish Core Facility, fed twice a day and maintained in a 14‐h light/10‐h dark cycle at 28.5°C, following the guidelines of the IBG Animal Care and Use Committee.

### 2.3 Zebrafish larval xenotransplantation

IM was excluded from any injection mixture to avoid pharmacological effects on the embryo and to focus only on cellular dynamics. For the metastasis or the population kinetics assays; 2 days post‐fertilization (dpf) embryo of Casper strains were dechorionated with pronase and anesthetized with 0.04 mg/mL tricaine (Sigma‐Aldrich) in embryo medium E3. K562 and K562‐IR cells were labelled with 2 mg/mL DiI (red) or DiO (green) lipophilic fluorescent dyes (# V2288, Thermo Fisher Scientific Inc., MA, USA) according to manufacturer's instructions. 50.000/μL cells in cell culture media were loaded into the borosilicate glass needle and 5–10 nL suspension containing 200 cells was injected into the yolk sac of 48 hpf embryos. The embryos were first examined for proper yolk sac injection, and any embryos showing cells in the bloodstream were excluded 24 h after injection. For the IM metastatic response assay, K562 cells were incubated with 20 μM (to obtain cell debris) and 5 μM IM (clinical median peak plasma concentration,^19^) for 72 h and cell viability was determined by haemocytometer using Trypan blue dye. Cells were then harvested by centrifugation and injected into the yolk sac of 2 dpf larvae. Control cells and 5 μM IM‐treated cells were centrifuged for 5 min at 1200 rpm and 20 μM IM‐treated cells for 15 min at 5000 rpm. Cells were then injected into the yolk sac of 2 dpf larvae and larvae were monitored at 3 days post‐injection (dpi)/5 dpf.

For the extravasation and the angiogenesis assays; 2 dpf larvae of Tg(fli1:EGFP) transgenic strains were injected with K562 or K562‐IR cells labelled with DiI (red). 50 cells and 100 cells were injected into the circulation and lateral perivitelline space (PVS), respectively. For the angiogenesis assay, the larvae were embedded in %3 methylcellulose with tricaine before the injection. The larvae that contain a visible cell mass at the desired area were collected and transferred to an incubator to maintain at 34°C for 2 days (4 dpf).

### 2.4 Image analyses and quantification

The invasiveness/metastasis and angiogenic potential assays were performed using fluorescence microscopy (Olympus IX71). Extravasation assays were performed using confocal microscopy (Zeiss LSM 880) with z‐stack and tile‐scan configurations. To prepare the larvae for confocal microscopy, live larvae were anesthetized and embedded in 1% low‐melting agarose in E3 medium. After visualization, larvae were recovered and placed back into the incubator for visualization the next day. Signs of extravasation were examined on the same larvae for three consecutive days. Red cells in each larva were counted and used to calculate the extravasated percentage of cells. The larvae in each experimental set were observed for the presence of motile cells and new vascular development.

### 2.5 Statistical analyses

Data were analysed using parametric or adequate non‐parametric statistical tests. Analyses were performed using GraphPad Prism v8. Statistical significance is indicated as *p < 0.05; **p < 0.01 and ***p < 0.001; ns stands for non‐significant. Exact numbers of the experiments were done in the Supplementary file.

## 3 RESULTS

### 3.1 K562‐IR resistant cells exhibit increasing invasive motility compared to the ancestor K562 cells

**FIGURE 1:**
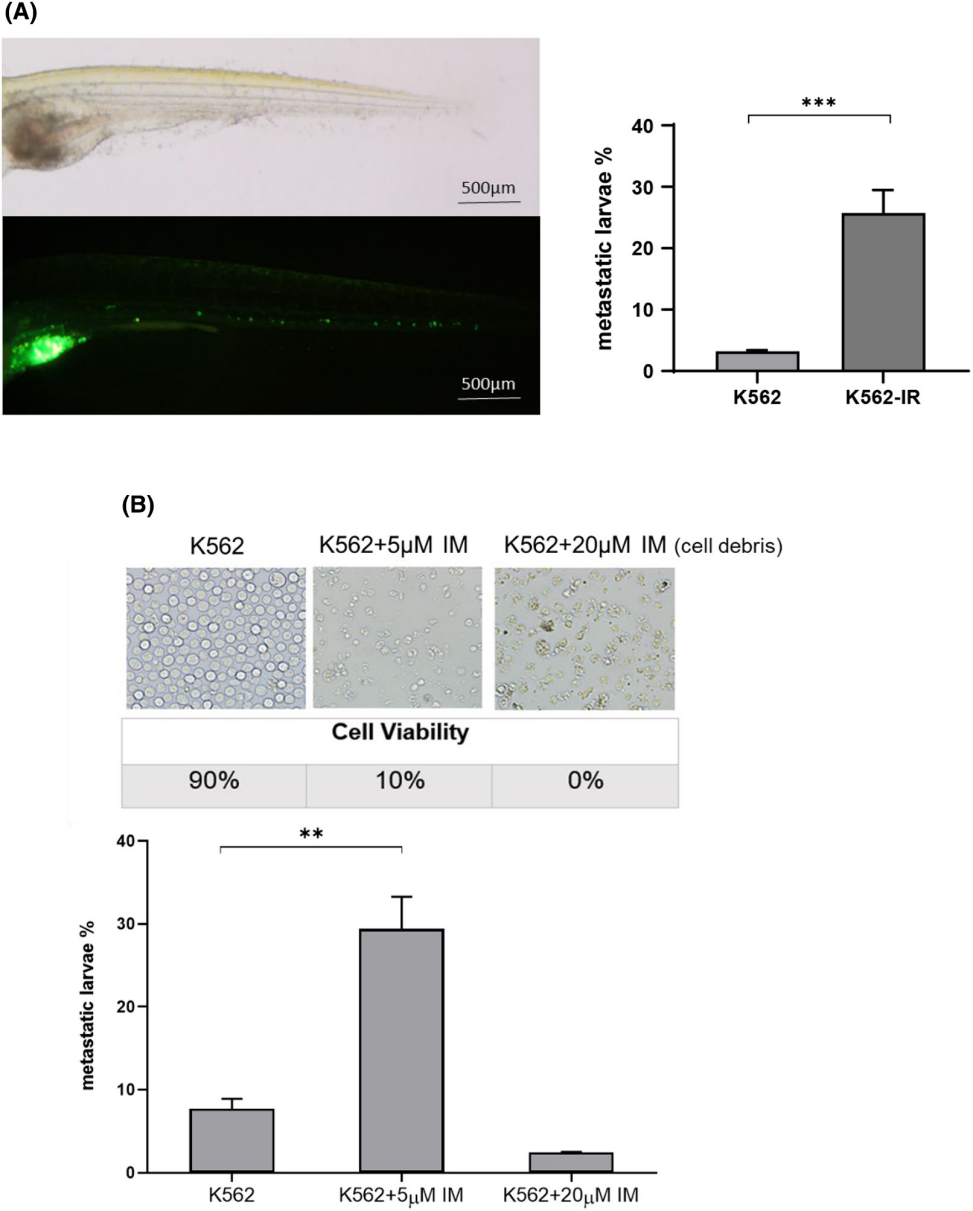
Invasiveness/motility capacity of K562 and K562‐IR cells. (A) Metastatic larvae example and metastatic larvae percentage graphic of K562 and K562‐IR cells is presented at 3 dpi (day post‐injection); ***p < 0.0005 (student *t*‐test). (*n* = 90 larvae/group) (B) IM effect on cellular invasiveness/motility in K562 cells. Metastatic larvae percentages graphic of the IM treatment groups is presented at 3 dpi; **p < 0.005 (student *t*‐test). K562 + 20 μM IM group is used as control. After 72 h treatment cell viability percentages were %90 for control cells, 10% for 5 μM IM‐treated cells and 0% for 20 μM IM‐treated cells. (*n* = min 60 larvae/group).

Unlike K562 cells, K562‐IR cells have spindle‐like morphology, are semi‐adherent and capable of forming tumour spheroids in three‐dimensional cell cultures. To determine and compare the invasiveness potential of the cells, we inoculated 100–200 stained K562 or K562‐IR cells separately into the yolk sac of 2 dpf larvae. At 5 dpf, the metastatic larvae percentage is monitored and calculated. The experiments were designed to have at least 90 larvae per group. We found that the motility of the K562‐IR cells is approximately seven‐fold higher than that of the K562 cells (Figure 1A, Supplementary file Table S1).

### 3.2 Exposure to IM contributes to the acquisition of cellular motility in sensitive leukaemic cells

Since the K562‐IR cells obtained by prolonged exposure to IM, we constructed an assay with sensitive K562 cells to explore the possible effect of IM on the motility behaviour of leukaemic cells. We first stained the K562 cells with fluorescent dye and treated with 20 μM (high dose), 5 μM (clinical median peak plasma concentration) and 0 μM IM in cell culture. The 20 μM high dose group (cell debris) was included to exclude the false‐positive migration of dead cells. Cell viability and metastatic larvae percentages are presented in Figure 1B (Supplementary file Table S2). These data show that surviving cells (10%) in the K562 population have a higher potential for metastatic/invasive progression. Imatinib mesylate has potential to contribute to cellular motility in leukaemic cells.

### 3.3 TKI‐ resistant K562‐IR cells show enhanced migratory potential within a mixed population of cells

**FIGURE 2:**
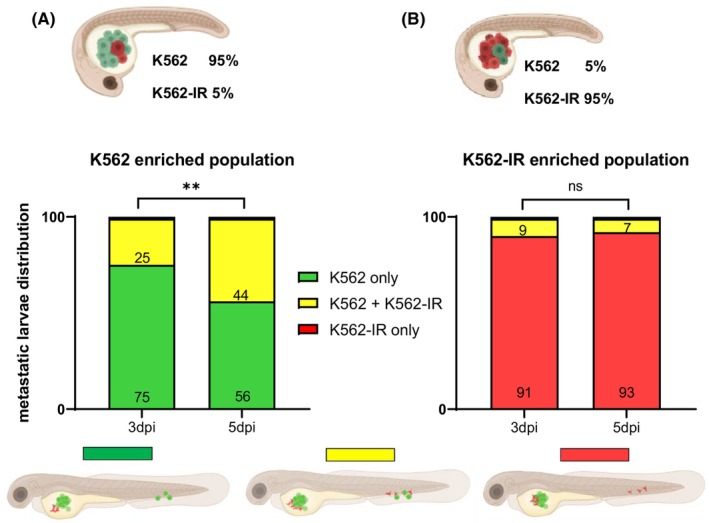
Comparison of metastatic capacity in a mix population assay (dpi: day post‐injection). Metastatic larvae distribution of the K562 cell enriched population and resistant K562‐IR cell population is presented as bar graphics in (A, B). To calculate the percentage of groups in the mixed populations, the total metastatic larvae number were normalized as 100%. Green bars show metastatic larvae with only K562 cells, red bars show metastatic larvae with only K562‐IR cells and yellow bars show metastatic larvae with co‐migrating K562 and K562‐IR cells together *n* = min.37 larvae/group; Chi‐square test **p = 0.0066.

To recapitulate the behavioural dynamics of a drug‐resistant and phenotypically altered subpopulation in CML patients, we labelled the IM‐sensitive and IM‐resistant cells with different vital dyes (K562 with DiO‐green; K562‐IR with DiI‐red) and mixed them in ratios of 5%–95% and 95%–5%. A mix of cells was injected into 2 dpf larvae. After identification of the larvae that harbour migrating cancer cells at 3 dpi, larvae containing only migrating K562 cells or K562‐IR cells as well as those harbouring both migrating cell types were counted separately at 3 and 5 dpi (Figure 2).

The motility of K562‐IR cells gradually increased from 25% to 44% between 3 and 5 dpi in the group where K562 cells are predominant (K562 95%‐K562‐IR 5%). Strikingly, despite the significantly smaller number of K562‐IR cells in the total population, their motility persisted noticeably (Figure 2A, Supplementary file Table S3). A slight co‐migration of K562 cells was observed in the group where K562‐IR cells are prevalent (K562 5%‐K562‐IR 95%). However, in K562 5%‐K562‐IR 95% group, the motility of K562 cells remained low and did not exhibit an increase over time (3 dpi‐7%; 5 dpi‐9%) (Figure 2B, Supplementary file Table S4). Since no larvae with only K562‐IR cells were observed in the K562 enriched group, and similarly, no larvae with only K562 cells were observed in the K562‐IR enriched group, these data are not presented in Figure 2A,B, respectively. When two groups were compared (K562 enriched vs. K562‐IR enriched graphics) at 3 dpi, the motility of the minority resistant K562‐IR cells was higher (25%) than that of the minority K562 cells (9%) in the mixed population. Overall, our data suggest that in a heterogeneous cancer cell population, where a small number of cells display phenotypic plasticity, there is a higher probability that these therapy‐resistant cells will migrate to distant locations.

### 3.4 K562‐IR cells exhibit a higher ability for extravasation and angiogenic potential

**FIGURE 3:**
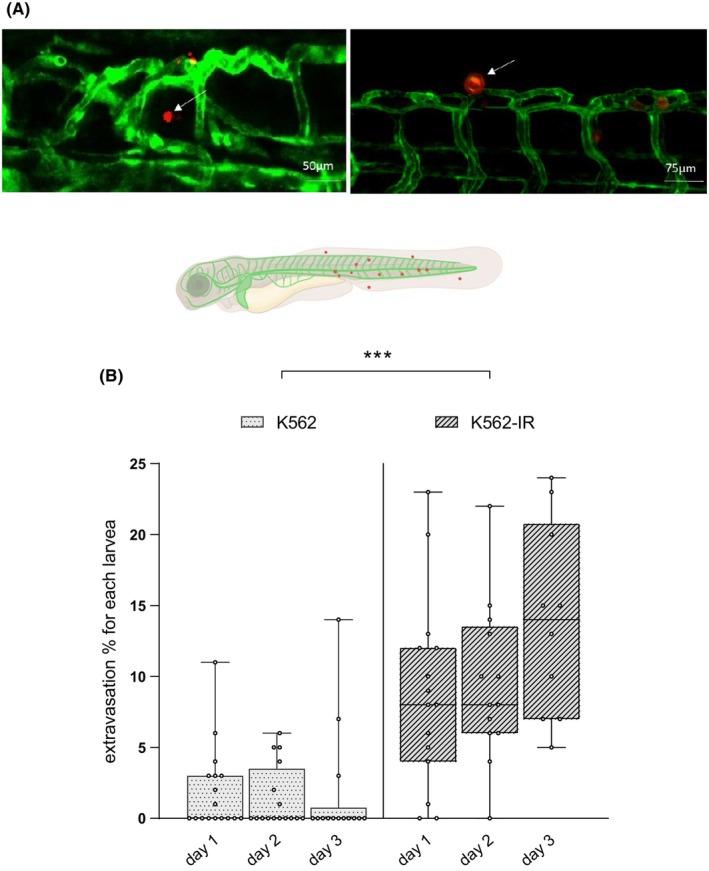
Extravasation ability of the K562 and K562‐IR cells. (A) Confocal microscopy z‐stack images showing the extravasated cells from the vasculature system of zebrafish larvae (trunk). (B) Extravasation observation assays were done with the same larvae for 3 days repeatedly. The percentage of extravasated cells was presented in box plot graphics. (*n* = 17 larvae/group; Wilcoxon test ***p value = 0.0001).

Given that K562 cells are derived from blood tissue components, we next aimed to investigate their potential for extravasation from the circulatory system. To this purpose, we outcrossed adult Casper mutants to the Tg(fli1:EGFP) transgenic strain of zebrafish and collected the embryos. To assess the ability of cells travelling through blood vessels to penetrate the somatic tissues, we injected K562 and K562‐IR cells directly into the circulatory system of the outcrossed embryos at 2 dpf. We employed confocal microscopy to monitor the progression of the injected cells and conducted a comprehensive examination on every cell present within the larvae, spanning from the yolk sac to the tail end. Specifically, we identified and quantified cells located in the extravascular tissue. Our findings suggest that K562‐IR cells exhibit an enhanced ability to extravasate from blood vessels into the surrounding tissue (Figure 3, Supplementary File Table S5).

**FIGURE 4:**
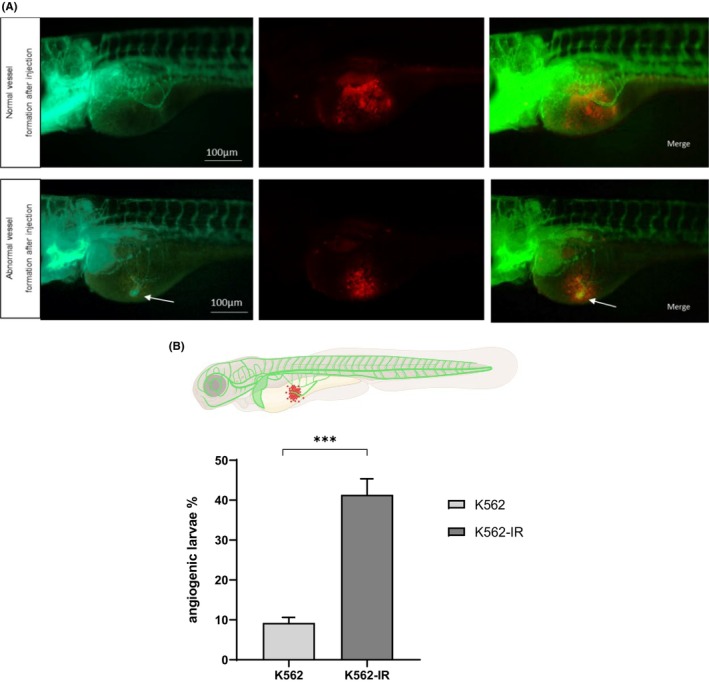
Angiogenic potential of the K562 and K562‐IR cells. (A) Representative examples of fluorescence microscopy images of larval xenografts at 2 dpi. The white arrows indicate a new vessel formation near the SIV. (B) Graph showing the percentage of larvae with abnormal/new vessel formation in the K562 and K562‐IR groups. (*n* = 90 larvae/group; student *t*‐test ***p = 0.0002).

Our previous gene expression profiling by mRNA microarray analyses revealed that K562‐IR cells differentially express vascular development genes.^4^ To examine the angiogenic capability of the TKI‐resistant and phenotypically plastic K562‐IR cells in vivo, we used the embryos obtained from the outcross of Casper mutant and Tg(fli1:EGFP) transgenic zebrafish. Following injection of DiI‐labelled K562 or K562‐IR cells into the lateral perivitellar cavity, near the subintestinal vessels (SIVs), we observed the larvae for their SIV morphology. Larvae injected with K562‐IR cells exhibited significantly increased new branch or node formation compared to the larvae having K562 cells, suggesting that K562‐IR cells promoted tumour‐related new blood vessel formation (Figure 4A,B, Table S6).

## 4 DISCUSSION

Phenotypic plasticity enables cancer cells to survive and evade the effects of anticancer therapies, posing a significant challenge in cancer treatment. By utilizing adaptive transcriptional changes cancer cells can switch between different states that display resistance to chemotherapy, targeted therapies or other anticancer strategies.^20^, ^21^, ^22^ They may enter a dormant state, slow down cell division and maintain a minimal metabolic rate, thus avoiding the lethal effects of the changing environment. The adaptive transcriptional landscape facilitates the development of resistance through the rewiring of signalling pathways. Cancer cells that adapt to targeted therapies can activate alternative pathways or acquire additional epigenetic alterations, allowing them to survive and continue proliferating despite ongoing therapy.^22^, ^23^, ^24^ A deeper understanding of these adaptive evolutionary trajectories and cellular dynamics leading to therapy resistance is needed.

Cancer involves complex interactions between cancer cells and their surrounding microenvironment. These interactions result in the emergence of distinct cellular adaptive states, which subsequently influence tumour growth, invasion, metastasis and response to therapy.^25^, ^26^, ^27^, ^28^ We have previously reported the K562‐IR cell line, which is independent of BCR‐ABL, as a valuable model for examining the underlying mechanisms of primary resistance in CML.^4^ Despite their haematological origin, K562‐IR cells display unique characteristics reminiscent of dormant tumour cells that survive after therapy and are responsible for the reappearance of cancer. These cells exist in a partially reprogrammed state, having lost their original identity and adopting a metamorphic condition to ensure self‐preservation. This study provides insights into the in vivo cellular behaviours exhibited by K562‐IR cells. A series of investigations in zebrafish larval xenografts have revealed that drug‐resistant K562‐IR cells have a notably higher migration capacity and an increased invasion propensity compared to the drug‐sensitive K562 cells.

To assess the impact of IM on cell invasion, we treated K562 cells with IM and subsequently injected the surviving cells into zebrafish larvae. Importantly, the motility of cells that endured IM exposure showed a statistically significant increase compared to untreated control cells. This finding suggests that these cells may have acquired the capability to invade surrounding tissues because of IM exposure, and/or IM may promote the emergence of an invasive subpopulation. Both scenarios merit further investigation. Furthermore, a limited number of metastatic larvae were detected within the suspension group, consisting entirely of cellular debris. Regarding this technical aspect, we would like to highlight the possibility of mechanical migration of cell debris in zebrafish xenograft assays.

During imatinib treatment, resistant cells can emerge in CML patients due to phenotypic shifting, resulting in the coexistence of imatinib‐sensitive BCR/Abl + leukaemic cells and an imatinib‐resistant subpopulation. To imitate this profile, we generated a population with a small proportion (5%) of resistant cells mixed with IM‐sensitive cells and observed their dominance in cell migration. Despite the low number of K562‐IR cells in the population, we found that, unlike sensitive cells, they retained their invasive properties. This observation may be associated with the recurrence of cancer in patients stemming from a small number of dormant cells that were resistant to therapy as a result of their phenotypic plasticity. These cells retain their biological properties including mobility and invasion.

In addition, zebrafish angiogenesis experiments revealed that K562‐IR cells were capable to inducing significantly more new vessel formation. In support of this observation, our previous transcript data showed distinct gene expression patterns in K562‐IR cells for gene groups associated with the gene ontology annotations ‘blood vessel development’, ‘vascular development’ and ‘blood vessel morphogenesis’.^4^ Our zebrafish angiogenesis experiments have further supported these data by demonstrating that K562‐IR cells had the ability to induce more significant new vessel formation. This aligns with their modelling of dormant cells that retain the capacity for angiogenesis leading to tumour recurrence. In the context of CML pathogenesis, several reports state an increase in angiogenic factors in CML patients that correlate with disease progression; and report that leukaemic cells can stimulate endothelial proliferation and neovascularization in the bone marrow of patients. Angiogenesis may be a less studied but important factor in disease development with the potential to be targeted in future therapeutic strategies.^29^, ^30^, ^31^, ^32^ When we compared the ability of leukaemic cells to migrate out of the bloodstream, we revealed that K562‐IR cells outperformed the others. These findings support the APS model, in which phenotypically dynamic cell populations may hide in somatic tissues and remerge under favourable conditions, possibly impacting cell motility and invasion capacity.

In conclusion, utilizing a zebrafish larval xenograft model, we demonstrated that phenotypically plastic cancer cells exhibit superior cellular dynamics compared to the cells they originate from. Understanding the dynamic behaviour of phenotypically plastic cancer cells and variations in their capacity to invade surrounding tissues, migrate and form metastases, requires comparable experiments with the original neoplastic mass. Integrating these information fosters a more comprehensive understanding of cancer development, progression and response to treatment. The intercellular dynamics among cancer cell subpopulations are of paramount importance in cancer, contributing to tumour heterogeneity, driving tumour progression, facilitating metastasis, inducing therapy resistance and giving rise to dormant clones. Understanding and targeting these dynamics hold great promise for the development of more effective cancer treatments.

## AUTHOR CONTRIBUTIONS

**Seda Baykal:** Conceptualization (lead); data curation (lead); formal analysis (equal); funding acquisition (lead); investigation (lead); methodology (equal); visualization (equal); writing – original draft (equal). **Zeynep Yuce:** Conceptualization (equal); formal analysis (supporting); supervision (supporting); writing – original draft (supporting). **Gunes Ozhan:** Methodology (equal); project administration (lead); supervision (equal); validation (equal); writing – original draft (supporting); writing – review and editing (equal).

## CONFLICT OF INTEREST STATEMENT

The authors declare no financial or competing interests.

## Supporting information


**Data S1:** Supporting Information.

## Data Availability

Data available in article supplementary material.
