# Supplementary material for: Phenotypically plastic drug‐resistant chronic myeloid leukaemia cell line displays enhanced cellular dynamics in a zebrafish xenograft model
Source: J Cell Mol Med. 2024 Oct 11;28(19):e70105. doi: 10.1111/jcmm.70105 (PMC11467800; doi:10.1111/jcmm.70105)
Supplement: Supplementary file 1 — Data S1: Supporting Information. [file JCMM-28-e70105-s001.docx]

Supplementary Informations:

| Groups | Total larvae number | Metastatic larvae number |
| --- | --- | --- |
| K562 | 33 | 1 |
|  | 30 | 1 |
|  | 30 | 1 |
| K562-IR | 30 | 9 |
|  | 29 | 7 |
|  | 26 | 6 |

Table 1: Metastatic larvae percentage of K562 and K562-IR cells (Figure 1A) exact numbers

| Groups | Total larvae number | Metastatic larvae number |
| --- | --- | --- |
| K562 | 35 | 3 |
|  | 29 | 2 |
| K562 +5µM IM | 28 | 9 |
|  | 30 | 8 |
| K562+20µM IM | 40 | 1 |
|  | 41 | 1 |

Table 2: Metastatic larvae percentage of IM treatment on K562 cells (Figure 1B) exact numbers

| 3dpi | K562 enriched population larvae | | |  |
| --- | --- | --- | --- | --- |
|  | K562+K562-IR (yellow) | K562 only (green) | K562-IR only (red) | Xenotransplanted total larvae |
|  | 0 | 7 | 0 | 30 |
|  | 3 | 3 | 0 | 30 |
|  | 1 | 2 | 0 | 9 |
| Total | 4 | 12 | 0 | 69 |
| Total metastatic larva number | **16** | | |  |
| Percentage | **25** | **75** | **0** |  |

| 5dpi | K562 enriched population larvae | | | |  |
| --- | --- | --- | --- | --- | --- |
|  | K562+K562-IR (yellow) | | K562 only (green) | K562-IR only (red) | Xenotransplanted total larvae |
|  | 10 | 10 | | 0 | 30 |
|  | 10 | 15 | | 0 | 30 |
| Total | 20 | 25 | | 0 | 60 |
| Total metastatic larvae number | **45** | | | |  |
| Percentage | **44** | **56** | | **0** |  |

Table 3: Metastatic larvae distribution of mix population Figure 2A exact numbers

| 3dpi | K562-IR enriched population larvae | | | |
| --- | --- | --- | --- | --- |
|  | K562+K562-IR (yellow) | K562 only (green) | K562-IR only (red) | Xenotransplanted total larvae |
|  | 1 | 0 | 7 | 30 |
|  | 0 | 0 | 3 | 15 |
| Total | 1 | 0 | 10 | 45 |
| Total metastatic larva number | **11** | | |  |
| Percentage | **9** | **0** | **91** |  |

| 5dpi | K562-IR enriched population larvae | | | |
| --- | --- | --- | --- | --- |
|  | K562+K562-IR (yellow) | K562 only (green) | K562-IR only (red) | Xenotransplanted total larvae |
|  | 2 | 0 | 26 | 37 |
| Total metastatic larva number | **28** | | |  |
| Percentage | **7** | **0** | **93** |  |

Table 4: Metastatic larvae distribution of mix population Figure 2B exact numbers. Total metastatic larvae number is normalized as 100%

|  | Extravasated K562 cell number | | | Extravasated K562-IR cell number | | |
| --- | --- | --- | --- | --- | --- | --- |
| Embryo | **Day 1** | **Day 2** | **Day 3** | **Day 1** | **Day 2** | **Day 3** |
| 1 | 3 | 4 | 3 | 8 | 13 | 13 |
| 2 | 0 | 0 | 0 | 0 | 6 | 7 |
| 3 | 3 | 5 | 0 | 23 | 15 | 20 |
| 4 | 0 | 0 | 0 | 0 | 4 | 10 |
| 5 | 3 | 0 | 0 | 0 | 10 | 24 |
| 6 | 11 | 6 | 14 | 13 | 0 | 15 |
| 7 | 6 | 1 | X | 12 | 8 | 7 |
| 8 | 1 | 2 | 0 | 4 | 14 | 23 |
| 9 | 4 | 5 | 7 | 0 | 7 | X |
| 10 | 0 | 0 | 0 | 5 | 8 | 5 |
| 11 | 2 | X | X | 9 | 10 | X |
| 12 | 0 | 0 | 0 | 6 | X | X |
| 13 | 0 | 0 | 0 | 10 | X | X |
| 14 | 0 | 0 | 0 | 20 | X | X |
| 15 | 0 | 0 | 0 | 12 | 22 | X |
| 16 | 0 | 0 | 0 | 1 | 6 | 15 |
| 17 | 0 | 0 | 0 | 8 | X | X |

Table 5: Extravasation ability of K562 and K562-IR graphic (Figure 3B) exact cell numbers. “X” represent dead larvae

| Groups | Total larvae number | Angiogenic larvae number |
| --- | --- | --- |
| K562 | 29 | 3 |
|  | 31 | 3 |
|  | 26 | 2 |
| K562-IR | 32 | 12 |
|  | 34 | 14 |
|  | 33 | 15 |

Table 6: Angiogenic larvae percentage graphic Figure 4B exact numbers
